# Ideological asymmetries in online hostility, intimidation, obscenity, and prejudice

**DOI:** 10.1038/s41598-023-46574-2

**Published:** 2023-12-15

**Authors:** Vivienne Badaan, Mark Hoffarth, Caroline Roper, Taurean Parker, John T. Jost

**Affiliations:** 1 Department of Psychology, American University of Beirut, Beirut, Lebanon; 2 Department of Psychology, New York University, 6 Washington Place, 5th Floor, New York, NY 10003 USA; 3 Center for Data Science, New York University, 726 Broadway, 7th Floor, New York, NY 10003 USA

**Keywords:** Human behaviour, Psychology

## Abstract

To investigate ideological symmetries and asymmetries in the expression of online prejudice, we used machine-learning methods to estimate the prevalence of extreme hostility in a large dataset of Twitter messages harvested in 2016. We analyzed language contained in 730,000 tweets on the following dimensions of bias: (1) threat and intimidation, (2) obscenity and vulgarity, (3) name-calling and humiliation, (4) hatred and/or racial, ethnic, or religious slurs, (5) stereotypical generalizations, and (6) negative prejudice. Results revealed that conservative social media users were significantly more likely than liberals to use language that involved threat, intimidation, name-calling, humiliation, stereotyping, and negative prejudice. Conservatives were also slightly more likely than liberals to use hateful language, but liberals were slightly more likely than conservatives to use obscenities. These findings are broadly consistent with the view that liberal values of equality and democratic tolerance contribute to ideological asymmetries in the expression of online prejudice, and they are inconsistent with the view that liberals and conservatives are equally prejudiced.

## Introduction

It is a common assumption in social science that, as Erikson and Tedin^1^ put it, “Conservatives consider people inherently unequal and worthy of unequal rewards,” whereas “liberals are egalitarian” (p. 69). Generations of philosophers, social theorists, and political scientists have argued that a fundamental, if not *the* fundamental, difference between ideologues of the left and right concerns egalitarianism: liberal-leftists prioritize social, economic, and political forms of equality, whereas conservative-rightists accept existing forms of hierarchy and inequality as legitimate and necessary, and perhaps even desirable (e.g.,^2–8^). A stronger commitment to equality and tolerance explains evidence that has accumulated over several decades that, on both implicit and explicit measures, political liberals express less hostility than conservatives toward a wide range of social groups that are frequent targets of prejudice in society^5,8–20^.

Recently, however, the longstanding idea that liberals are more egalitarian, more tolerant, and less prejudiced than conservatives has come under attack. It has been argued that liberal-leftists are every bit as authoritarian, intolerant, and hostile toward dissimilar others as are conservative-rightists^21–26^. The overarching claim is that leftists and rightists are equally biased, but they are just biased against different groups^27^. There is also an untested assumption in the literature on “worldview conflict” and prejudice^27^ that conservatives are biased against Black people and women not because of race or gender, but merely because they assume that Black people and women are liberal. Thus, whereas rightists are said to express prejudice against groups that are presumed to be left-leaning (such as Black people, atheists, and women), leftists are said to express prejudice against groups that are presumably right-leaning (such as businesspeople, Christians, and men).

The vast majority of evidence put forward on behalf of the ideological symmetry perspective is based on self-reported attitudes, such as feeling thermometer ratings of how “cold” or “warm” people feel toward specific target groups. A typical, albeit unsurprising finding is that social conservatives feel more warmth toward groups perceived as socially conservative (vs. liberal), whereas social liberals feel more warmth toward groups perceived as socially liberal^28^. However, we think that there are several major problems with investigating ideological symmetries and asymmetries in prejudice this way^29^.

To begin with, most of the research purporting to document ideological symmetries in prejudice merely shows that liberals and conservatives sometimes express lukewarm attitudes toward specific groups. This body of work relies upon what we consider to be a watered-down definition of prejudice as any “negative evaluation... on the basis of group membership,” which “does not depend on whether such a prejudice can be justified according to some moral code” (p. 359)^30^. This conceptualization departs radically from “classic” definitions of prejudice in social psychology, such as Gordon Allport’s^31^ treatment of prejudice as “thinking ill of others without sufficient warrant” (p. 6), that is, “an antipathy based upon a faulty and inflexible generalization... directed toward a group as a whole, or toward an individual because he is a member of that group” (p. 9). Textbook definitions likewise emphasize “a hostile or negative attitude toward a distinguishable group based on generalizations derived from faulty or incomplete information” (p. 231)^32^, and “an unjustifiable (and usually negative) attitude toward a group and its members [involving] stereotyped beliefs, negative feelings, and a predisposition to discriminatory action” (p. G–10)^33^. When social scientists seek to understand and ameliorate prejudice, we expect that they are not concerned merely with the expression of lukewarm attitudes but with the kind of intense, unwarranted negative affect that motivates hostility, hatred, intimidation, and discrimination (e.g.,^34^).

To overcome limitations of previous research on the subject, and to investigate the hypothesis that liberal commitments to equality and democratic tolerance would contribute to an ideological asymmetry in expressions of hostility, intimidation, and prejudice, we conducted a large-scale investigation of naturally occurring social media behavior. Specifically, we harvested a large corpus of Twitter messages based on keywords that included social groups that, according to previous research, are common targets of liberal prejudice (e.g., Catholics, Whites, wealthy people, and conservatives) and conservative prejudice (e.g., Blacks, illegal immigrants, and liberals). In addition, we implemented a Bayesian Spatial Following model to estimate the ideological positions of Twitter users in our sample, so that we could compare the online behavior of left- and right-leaning social media users. Finally, we used a combination of manual and automatic text-coding methods to investigate ideological asymmetries in the use of language containing (1) threat and intimidation, (2) obscenity and vulgarity, (3) name-calling and humiliation, (4) hatred and racial, ethnic, or religious slurs, (5) stereotypic generalizations, and (6) negative prejudicial language. We hypothesized that: (HI) tweets mentioning liberal- or left-leaning target groups will contain more expressions of online prejudice than tweets mentioning conservative- or right-leaning target groups; and (HII) tweets sent by conservative- and right-leaning users will contain more expressions of online prejudice than tweets sent by liberal- and left-leaning users.

## Method

### Data collection and inclusion criteria

We used a supervised machine-learning approach to analyze naturally occurring language in a very large number of social media posts sent by liberal-leftists and conservative-rightists in reference to groups that have been identified as likely targets of liberal and conservative bias. The population of interest was the set of messages circulated in the U.S. Twittersphere. Between March and May 2016, we harvested 733,907 Twitter messages that included one or more of the 96 keywords listed in Table 1, including *progressives, rightists, Christians, civil rights activists, Caucasians, Black people, destitute*, and *rich people*. The selection of target groups was based on previous research by Chambers et al.^23^ and Brandt et al.^22^, which sought to specify frequent targets of “liberal prejudice” and “conservative prejudice.” For each of the target groups, we included synonyms, all of which were either hashtags or keywords used on Twitter during the period of data collection. All search terms were manually inspected prior to data collection. Some of the terms were deemed by the computer scientists implementing the queries as too common on Twitter to be included in the collection, so they were excluded. To filter out tweets that contained pornographic content and those written in languages other than English, respectively, we included pornography and non-English as categories in the human coding and machine-learning phases. We excluded tweets that, through machine-learning classification, had a probability of containing pornographic content greater than 0.50 and being non-English greater than 0.50. This left us with a total sample of 670,973 tweets that were eligible for further analysis.

**Table 1:** Keywords used to harvest tweets for the data collection.

Liberal target groups		Conservative target groups	
Target group	Alternative keywords	Target group	Alternative keywords
Liberals	Progressives Left-wing	Conservatives	Right-wing Rightists Fascists
Radical students	Radicals Student activists	Middle class people	The middle class Middle income
Atheists	Godless Non-believers	Whites	Caucasian Crackers
Gays and lesbians	LGBT/LGBTQ Homosexuals Queer Transgender	Protestants	Methodists Lutherans Pentecostal Presbyterian Adventist
Labor unions	Unions Trade unions	Elderly people	The elderly Senior citizens
Illegal aliens	Immigrants Immigration Undocumented Refugees Refugee crisis	Wealthy people	Rich The 1% Trust fund babies Spoiled
People with AIDS	HIV-positive	Christian fundamentalists	Fundamentalists Christians Evangelicals Baptists
Environmentalists	Tree-huggers Hippies Greens	Anti-abortionists	Pro-life Anti-choice
Civil rights leaders	Social justice warriors (SJW) AntiSJW Community organizers Civil rights activists	Military	Soldiers Veterans Armed forces The Navy Army Marines Coast guard
Blacks	African American Black people Black History Month N******	Business people	Businessmen Executive Capitalist
Poor people	Destitute Impoverished Underprivileged		
Young people	Millennials Teenagers		
Chicanos/Hispanics	Chicanas Latinos Latinas		
Asian Americans	Asians		

### Ideological estimation

We used Barberá’s method of estimating left–right (or liberal-conservative) ideological positions of Twitter users^36^. This method, which has been validated in a number of ways, employs a Bayesian Spatial Following model that treats ideology as a latent variable estimated on the basis of follower networks, that is, the number of liberal and conservative political accounts (of well-known journalists, politicians, and other political actors) that the individual follows. We were able to calculate point estimates for a total of 325,717 Twitter users. Scores ranged from -2.611 (very liberal) to 4.668 (very conservative), with a mean of 0.369 (SD = 1.724). The mean indicated that, on average, the users in our sample were moderate (neither liberal nor conservative). Using this method, 176,948 Twitter users in our sample were classified as liberal-leaning (that is, below zero), and 148,769 were classified as conservative-leaning (above zero).

### Human coding phase

To train the automatic machine-learning algorithm to classify tweets, it was necessary to first have a subset of them manually coded. Before rating the tweets that were used for the machine learning phase, all raters participated in a two-hour training session and were taught to follow the same standardized protocol (see Human Coding Manual in Supplementary Material). In the pilot coding phase, seven trained research assistants coded a total batch of 1000 tweets (500 tweets each) to assess the appropriateness of the coding instructions. We then used their feedback to make clarifications, minor revisions, and edits to the coding manual. In the next phase, 11 trained undergraduate and graduate psychology students coded an additional set of 6000 tweets. The final sample of manually coded tweets therefore consisted of *N* = 7000 unique tweets, with each tweet coded by at least three independent raters.

#### Coding categories

To establish our coding scheme, we conducted an extensive literature search on studies of online incivility and the linguistic expression of prejudice. Incivility in online discourse is operationally defined in terms of the use of disrespectful language^37,38^. Disrespectful language can be broken down further into the use of obscene language and name-calling or attempts to humiliate the target of the disrespectful language. In the context of intergroup relations, incivility may also include the use of aggressive, threatening, or intimidating language. Because a main goal of our research program was to investigate ideological symmetries and asymmetries in prejudice, we estimated the prevalence of negative prejudicial language, which is underpinned by stereotypical categorical generalizations expressed in a way that renders them largely immune to counterevidence^11,17,31,34,35^. Thus, we sought to analyze prejudicial language directed at specific target groups that are typically perceived to be left- and right-leaning, respectively. Because our dataset was harvested before Twitter expanded its policies against hate speech and hateful conduct in late 2019, we were able to investigate hatred directed at various target groups.

Therefore, research assistants coded the tweets on all of the following dimensions: (1) Threat/intimidation: language conveying a threat to use physical violence or intimidation directed at an individual or group; (2) Obscenity: an offensive word or phrase that would be considered inappropriate in professional settings; (3) Hatred: a communication that carries no meaning other than the expression of hatred for some social group; (4) Name-calling/humiliation: language directed at an individual or group that is demeaning, insulting, mocking, or intended to create embarrassment; (5) Stereotypic generalization: false or misleading generalizations about groups expressed in a manner that renders them largely immune to counterevidence; and (6) Negative prejudice*:* an antipathy based on group-based generalizations, that is, an unfavorable feeling “toward a person or thing, prior to, or not based on, actual experience” (p. 6)^31^.

Inter-rater reliability coefficients for each of these categories are provided in the Online Supplement (Tables S.1–S.8). We used a majority voting method, so that if two or more of the three human coders agreed that a given tweet contained hatred, obscenity, prejudice, and so on, it was classified as belonging to the positive class. Coding frequencies estimated for the training data set are summarized in Table S.9 of the Supplement for each of the six theoretical categories (plus the two screening categories).

### Machine-learning phase

Training, validation, and test sets for the machine-learning phase were based on the 7000 human-coded tweets. We reserved 20% (1400) of the tweets to use as a test set to evaluate final model performance. Of the other 5600 tweets, 20% (1100) were used for purposes of validation, leaving 4500 tweets with which to train the models. We used several different text classification strategies, including “bag of words” models such as the Support Vector Machine (SVM), neural networks such as Long Short-Term Memory (LSTM), and transfer learning techniques such as Universal Language Model Fine-Tuning (ULMFiT) and Bidirectional Encoder Representations from Transformers (BERT). We applied each of these strategies to classify the tweets according to the six dimensions of classification. For the sake of brevity, we report results from the best performing model, namely BERT. Detailed information about all machine-learning methods and results are provided in the Online Supplement, along with a comparative analysis of the four machine learning models employed.

#### Bidirectional encoder representations from transformers

BERT is an innovative state-of-the-art language representation model^39^. Developed by researchers at Google AI Language, BERT creates a “deep bidirectional representation” of language, which means that the representation of the language is contextualized, with each word conditioned on the preceding and succeeding words. A traditional language model is built by optimizing an objective function that seeks to accurately predict the next word, given the preceding context. BERT instead randomly “masks" words and seeks to predict the masked word given the language that precedes and succeeds it.

BERT uses units called *transformers*, as originally implemented by^40^. The transformer is an alternative to convolutional and recurrent architectures that builds on the concept of multi-head attention. Traditional attention mechanisms in sequence-to-sequence models establish a correspondence between units of the input and units of the output. Multi-head attention can relate parts of a single sequence to each other, within either the input or the output. The BERT model also represents language as word parts, not just full word tokens. So, for example, it divides the word “mongering” into “mon,” “ger,” and “ing.” This use of bipartite encodings of words is common in NLP research, but it is especially important when analyzing Twitter data, which often contains misspellings and abbreviations. In addition, Twitter hashtags are often comprised of several words combined without a space, so tokenizing only on words properly divided by spaces would be potentially problematic.

To implement our version of the BERT model, we used the publicly available PyTorch code. Although the original authors of BERT used TensorFlow, they have formally endorsed the PyTorch implementation, and experiments have verified that it produces identical results^41^. We started from the publicly available BERT model, pre-trained on the BooksCorpus (800 M words) and English Wikipedia (2500 M words). There are two publicly available versions of the BERT model. The large version has 16 attention heads and 24 layers, whereas the base version has 12 attention heads and 12 layers.

## Results

### BERT machine learning model

The results for tuning the BERT model are shown in Table 2. The creators of BERT recommend experimenting with batch sizes of 16 and 32, learning rates of 5e^−5^, 3e^−5^ and 2e^−5^, and epochs 3 and 4. We ran 6 of the 12 possible combinations, and also experimented with choosing a smaller batch size and learning rate than BERT’s authors would typically recommend. All results described below are based on the large pre-trained BERT model. An undefined *F*-score occurs when no correct positive class predictions are made. Because our classes were highly imbalanced, this usually indicates that the model did not predict any positive incidences. The tuning results indicated that 3 epochs, a learning rate of 2e^−5^, and a batch size of 16 performed well. However, when we ran this tuned model on the other category labels, we encountered several degenerate results by using the “large” model on a small dataset. Obscenity, name-calling, negative prejudice, and non-English all produced undefined *F*-scores. The creators of BERT overcame the problem of degenerate results by experimenting with several random initializations until one version succeeded. We instead examined validation scores using the same parameters on the “base” version of the model.

**Table 2:** Validation F-scores from the BERT Model.

Label	*f-*score
Obscenity	0.741
Hatred	0.700
Name calling	0.553
Negative prejudice	0.493
Threat	0.423
Stereotypes	0.330

### Hypothesis testing

In Table 3 we display the number and percentage of all tweets that, according to machine-learning analyses, contained each of the categories of linguistic bias. Here we define a tweet containing a positive instance as that with *p* (category) > 0.50. Negative prejudice—the expression of hostile or unfavorable attitudes on the basis of categorical group membership—was present in 13.0% of the tweets in our sample (*N* = 87,250). Hateful speech was the least common category, with 2.20% of the Tweets (*N* = 14,690) containing positive instances.

**Table 3:** Number and percentage of tweets containing positive instances of each linguistic category according to machine-learning analyses of the complete data set.

Linguistic category	*N*	Percentage of total tweets
Hatred	14,690	2.20
Threat	23,975	3.60
Obscenity	29,908	4.50
Name-calling	52,823	7.90
Stereotypes	46,556	6.90
Negative prejudice	87,250	13.0

#### Target group effects

We hypothesized that messages referring to liberal or left-leaning target groups would contain more indicators of linguistic bias than messages referring to conservative or right-leaning target groups. Because it was not necessary to restrict this analysis to messages sent by users for whom we were able to classify their ideological position, we conducted this analysis based on the larger sample of 670,973 tweets. The perceived ideological leanings of the various target groups were estimated based on data from Chambers et al. (Sample 1)^22^, as graphed by Brandt et al. (Fig. 2)^21^.

Target ideology scores ranged from 1.29 (very liberal) to 4.65 (very conservative), with a mean of 2.876 (SD = 1.108). As hypothesized, target ideology was significantly and negatively associated with each of the linguistic bias categories (see Table 4). That is, the more liberal/leftist the target group was perceived to be, the more likely it was for tweets mentioning that group to contain hatred, threatening language, obscenity, name-calling, stereotyping, and negative prejudice. Most of the correlations were relatively small, but all were statistically significant at *p* < 0.001. The two largest effect sizes were for name-calling (*r* = −0.146) and the expression of negative prejudice (*r* = −0.126).

**Table 4:** Bivariate correlations between target ideology (groups that were perceived as more conservative/rightist) and the expression of linguistic bias overall (*N* = 670,973 tweets).

	1	2	3	4	5	6
1. Target ideology	–					
2. Hatred	− .070***	–				
3. Threat	− .055***	.125***	–			
4. Obscenity	− .063***	.675***	.065***	–		
5. Name calling	− .146***	.457***	.100***	.456***	–	
6. Stereotypes	− .098***	.221***	.178***	.196***	.402***	–
7. Negative prejudice	− .126***	.339***	.215***	.330***	.621***	.679***

Entries are Pearson’s *r* correlation coefficients. ***p < .001. Greater scores on target ideology indicates more conservative targets. As such, positive correlations indicate a greater presence of an attribute in tweets with conservative targets, and negative correlation indicate a greater presence of an attribute in tweets with liberal targets.

#### Communicator effects

Next, we investigated the effects of user ideology on linguistic bias. This analysis was based on the subset of messages (*n* = 325,717) sent by users who could be classified as liberal or conservative. As shown in Table 5, conservative Twitter users were more likely than liberal Twitter users to communicate negative prejudice (*r* = 0.210), name-calling (*r* = 0.146), stereotypes (*r* = 0.110), and threatening language (*r* = 0.092), all *p*s < 0.001. Conservatives were slightly more likely to use hateful language (*r* = 0.011), whereas liberals were slightly more likely to use obscenity (*r* = −0.010); both of these effects were quite small but, because of the very large sample size, still significant at *p* < 0.001.

**Table 5:** Correlations between user ideology (twitter users who were classified as more conservative/rightist) and the expression of linguistic bias, both overall and against specific target groups.

	Total Sample (*N* = 325,717)	Messages Mentioning Left-Leaning Groups (*n* = 229,788)	Messages Mentioning Right-Leaning Groups (*n* = 95,929)
Hatred	.011***	.021***	− .026***
Threat	.092***	.123***	.021***
Obscenity	− .010***	.003	− .047***
Name calling	.146***	.191***	.025***
Stereotypes	.110***	.116***	.096***
Negative prejudice	.210***	.247***	.118***

Entries are Pearson’s *r* correlation coefficients.

*** p < .001.

#### Communicator effects analyzed separately for liberal versus conservative target groups

Next, we inspected correlations between user ideology and linguistic bias directed at groups that were generally perceived to be liberal or left-leaning vs. conservative or right-leaning, respectively (see Table 5). For the subsample of tweets that mentioned liberal-leftist groups (*n* = 229,788), which comprised 70.5% of the total number of tweets in our collection, users who were classified as more conservative were more likely to express negative prejudice (*r* = 0.247), to engage in name-calling (*r* = 0.191), and to include threats (*r* = 0.123), stereotypes (*r* = 0.116), and hatred (r = 0.021), all *p*s < 0.001. There was no effect of user ideology on the use of obscenity (*r* = 0.003, *p* = 0.119).

For the much smaller subsample of tweets that mentioned conservative-rightist groups (*n* = 95,929), more liberal users were slightly more likely to express obscenity (*r* = −0.047) and hatred (*r* = −0.026), both *p*s < 0.001. However, for the remaining categories, conservative Twitter users were actually more likely than liberal Twitter users to express linguistic bias. That is, even when writing about groups that are generally considered to be right-leaning, conservatives were more likely to communicate negative prejudice (*r* = 0.118), stereotypes (*r* = 0.096), name-calling (*r* = 0.025), and threatening language (*r* = 0.021), all *p*s < 0.001.

### Sensitivity analyses

We conducted additional sensitivity analyses to determine whether the results and their interpretation was impacted by analytic decisions. Specifically, we re-coded the continuous estimates for linguistic bias into binary, categorical variables (< 50% probability = does not contain biased language, ≥ 50% probability = does not contain biased language) and conducted regression analyses. Results were very similar to those described above.

Tweets that mentioned liberal-leaning groups were more likely to contain hatred (*b* = − 0.45, *SE(b)* = 0.008, Wald = 2833.26), threats (*b* = − 0.26, *SE(b)* = 0.006, Wald = 1697.99), obscenity (*b* = − 0.23, *SE(b)* = 0.006, Wald = 1710.62), name calling (*b* = − 0.36, *SE(b)* = 0.004, Wald = 6783.19), stereotypes (*b* = − 0.22, *SE(b)* = 0.004, Wald = 2480.92), and negative prejudice (*b* = − 0.272, *SE(b)* = 0.003, Wald = 6386.04), all *p*s < 0.001.

We also compared the frequencies (percentages) of messages about various target groups that contained each type of linguistic bias. Tweets about left-leaning (vs. right-leaning) groups were again more likely to contain hatred (3.5% vs. 0.5%, *χ*^2^ = 6882.25), threats (4.1% vs. 2.9%, *χ*^2^ = 749.48), obscenity (5.8% vs. 2.8%, χ^2^ = 3521.28), name calling (10.4% vs. 4.8%, *χ*^2^ = 7342.45), stereotypes (7.9% vs. 5.7%, *χ*^2^ = 1254.16), and negative prejudice (15.4% vs. 10.1%, *χ*^2^ = 4205.00), all *p*s < 0.001.

Finally, we examined whether user ideology was related to the percentage of messages containing linguistic bias. Tweets sent by more conservative users had a higher probability of containing hateful language (*b* = 0.049, *SE(b)* = 0.008, Wald = 35.76), threats (*b* = 0.225, *SE(b)* = 0.005, Wald = 2055.62), name calling (*b* = 0.210, *SE(b)* = 0.003, Wald = 3766.95), stereotypes (*b* = 0.134, *SE(b)* = 0.003, Wald = 1475.32), and negative prejudice (*b* = 0.26, *SE(b)* = 0.003, Wald = 9125.68), all *p*s < 0.001. There was no statistically significant effect of user ideology in the use of obscene language (*b* = − 0.007, *SE(b)* = 0.006, Wald = 1.81, *p* = 0.179).

### Ideology of the coders

Because we were concerned that the political orientations of the raters could bias their coding, we asked the research assistants to answer three questions about their general political orientation (“Please indicate on the scale below how liberal or conservative [in terms of your general outlook] you are”), social attitudes (“How liberal or conservative do you tend to be when it comes to social policy?”), and economic attitudes (“How liberal or conservative do you tend to be when it comes to economic policy?”). Responses could range from 1 (*very liberal*) to 7 (*very conservative*). The 8 (of 11) raters who answered these questions were liberal leaning on average, *M* = 2.46 (*SD* = 1.05).

We examined point-biserial correlations between coders’ ideology scores and their rating of each linguistic category under study for every batch of tweets. We found that rater ideology was unrelated to the criterion linguistic category used to train the machine learning algorithm, i.e., hateful language (*r* = 0.009, *p* = 0.139). Rater ideology was also unrelated to the detection of threatening language in the training tweets (*r* = 0.011, *p* = 0.079). At the same time, the more conservative our raters were, the more likely they were to detect obscenity (*r* = 0.022, *p* < 0.001), whereas the more liberal our raters were, the more likely they were to detect name-calling (*r* = − 0.028, *p* < 0.001), stereotypes (*r* = − 0.136, *p* < 0.001), and negative prejudice (*r* = − 0.111, *p* < 0.001). Thus, coder ideology was inconsistently related to the use of various coding categories. Most importantly, ideology of the raters was unrelated to their ratings of hatred, which was used as the base linguistic model for training the other categories. It is also worth highlighting the fact that the classification and labeling process for the machine learning training relied on majority voting, so that at least two annotators must have agreed that the tweets contained hatred, obscenity, etc., before it was labeled as belonging to the positive class.

## General discussion

### Summary of findings and their implications

In this study, we investigated the question of whether online prejudice is symmetrical or asymmetrical on the political left and right in the U.S. in a very large sample of social media messages. We observed that Twitter messages mentioning targets perceived as liberal or left-leaning (such as Black Americans and feminists) included higher levels of hate speech, threat, obscenity, name-calling, stereotyping, and negative prejudice, compared to Twitter messages mentioning targets perceived as conservative or right-leaning (such as conservatives and Christians). These results supported (HI).

We estimated user ideology scores based on Barberá’s method^36^ and observed that whereas liberal users were slightly more likely than their conservative counterparts to use obscene language, conservatives were more likely to use negative prejudice, name-calling, and hateful and threatening language, although the effect sizes for the last two categories were very small. Perhaps the most important finding is that conservatives were more likely than liberals to use negative prejudicial language, and that negative prejudice was expressed more strongly in tweets mentioning purportedly left-leaning targets than in tweets mentioning right-leaning targets. These results are clearly consistent with (HII) and inconsistent with the alternative hypothesis that prejudice is symmetrical on the left and right^21–28,42^. Instead, they reinforce the long-standing, empirically supported conclusion that out-group prejudice is more prevalent on the right than the left^9–15,17–19,29^.

Because we measured the spontaneous use of language in a naturally occurring “real-world” setting, our results go well beyond what can be concluded based on studies using feeling thermometer measures of prejudice, which are subject to norms of socially desirable responding (for a critique of previous research in this area, see^29^). Our findings are also consistent with two other major studies of prejudicial outcomes in society. First, an analysis of FBI hate-crime data from 1996 to 2018 revealed that ostensibly left-leaning targets such as racial, religious, and sexual minorities were subjected to much higher levels of hate crime than ostensibly right-leaning targets, such as racial, religious, and sexual majorities^29^. Thus, group-based discrimination, which is an obvious manifestation of out-group prejudice, disproportionately affects disadvantaged target groups who are perceived as left-leaning in political orientation. Second, a comprehensive study of political violence carried out in the US between 1948 and 2018 showed that individuals who were affiliated with left-wing extremist movements had 68% lower odds of engaging in violent behavior, compared to individuals affiliated with right-wing extremist movements^13^. Thus, in these previous investigations, and in our present study, rightists were much more likely to be perpetrators of prejudice, and leftists were much more likely to be victims of prejudice. This is consistent with the view that substantial left–right ideological asymmetries exist when it comes to the thoughts, feelings, and behaviors of individuals and the social groups to which they belong (see^5^).

### Strengths and limitations

One strength of the present research program, which we alluded to above, is that it is high in external validity. This is because we unobtrusively observed the spontaneous language used by liberals and conservatives in actual social media communications referring to target groups that are perceived as left-leaning vs. right-leaning. Furthermore, by observing the expression of prejudice in vivo, focusing on naturally produced language, we avoided several common methodological artifacts that frequently hamper social psychological research on bias and prejudice, such as problems of experimenter bias and socially desirable responding. Another advantage of this study is that the final sample size of messages analyzed was very large (*N* = 670,973), rendering our estimates both highly stable and robust.

Yet another strength of our study is that we used cutting-edge machine learning methods in data science to investigate social psychological hypotheses and, in particular, to classify linguistic phenomena, such as the expression of negative prejudice, that have historically been very difficult to classify using objective methods. In the process of developing our computational model, we generated a set of 7000 labelled tweets that is available for future researchers to train their own machine learning models. All of these tweets were rated by three different human coders, so that we could ensure high levels of interrater reliability before training our various machine-learning algorithms. Although the procedure was both time- and resource-intensive, it increased the accuracy of predictions made by the machine-learning models. We have emphasized results based on the best-performing algorithmic model (BERT) in this article, but the data scientists on our team tested and fine-tuned four different classification models. The methods and results associated with these other algorithms are described in the Online Supplement.

Of course, this study also has its limitations. For one thing, the Twitter API limited the number of data queries we were able to submit during the period of data collection, which means that the dataset does not include all potentially relevant tweets sent during the period of investigation. However, we were able to collect a random sample of the total population of tweets sent during the period in question. The Twitter messages we harvested were from March to May of 2016. This was before the primary and presidential elections of 2016, which means that it was prior to Donald Trump’s nomination and eventual election to the presidency. Given the intensity of Trump’s public rhetoric against many of the left-leaning target groups listed in Table 1 (especially immigrants, racial minorities, liberals, and leftists), and the uptick in hate crimes and other cases of prejudice and discrimination that accompanied his presidency, e.g., see^43–46^, the timing of our investigation means that we may have underestimated the true extent of online bias and harassment committed by rightists against target groups that are perceived as left-leaning in the period that immediately followed our investigation.

Another technical limitation concerns the performance of our optimal machine learning algorithm. Although the algorithm had high *f-*scores with respect to hatred, obscenity, and name calling, it performed less than optimally with respect to the categories of negative prejudice, threat, and stereotyping. This could be attributable to (a) the difficulty in detecting relatively “fuzzy” concepts; (b) the fact that our operationalization of stereotypes included all group-based generalizations, not only *negative* group-based generalizations; and/or (c) an insufficient amount of training data, although the research team coded as many tweets as was logistically feasible giving timing and other constraints. Future research would do well to overcome these limitations by (a) using sentiment analysis to code the valence of the attitudes in the tweets; (b) focusing exclusively on negative stereotypes; and (c) annotating a larger corpus of training tweets. Despite the limitations of our study, we believe that it is the first of its kind to use robust machine-learning models to assess multiple indicators of online prejudice.

As in every other study of social media communication, our analysis is highly dependent upon the selection of keywords and search terms used to construct the data set. We first selected social groups based on previous research to identify potential targets of “liberal prejudice” and “conservative prejudice” and then generated synonyms for those groups^22,23^. However, some words and phrases (such as “Democrats” and “Republicans”) were determined by our computer technicians to occur too frequently in the total population of tweets; these were dropped to make the data collection more manageable. Although this did introduce some degree of selectivity in the search terms used, we note that the data set is based on 96 words and phrases, which is an extremely large sample of keywords compared to other studies of online hostility and prejudice.

The non-experimental study design prohibits the drawing of causal conclusions about the nature of ideology and prejudice. Moreover, there are several third variables—such as intelligence, education, authoritarianism, social dominance orientation, system justification, and the like—that may help to explain *why* conservative-rightists express more online prejudice than liberal-leftists (e.g., see^5,8–10,12,47,48^). Future research would do well to measure these as mediating or moderating variables.

The fact that our analyses are confined to a single social media platform is yet another limitation. Because Twitter changes its policies regarding the removal of potentially prejudicial content every few years, our analysis was bounded by their terms of service during the period of data collection. According to the results of a Pew Survey in 2021, Twitter users tend to be younger and more Democratic, compared to the public at large. Therefore, although our sample is much larger and more representative of the general population than in studies of prejudice based on convenience samples, we do not know how well these results would generalize to the population of U.S. adults.

It would be useful to conduct parallel studies about the role of political ideology in the expression of prejudice on other platforms, such as Facebook, Instagram, and Reddit, as well as social media channels that are favored by right-wingers, such as 4chan, Parler, and Trump’s own social media platform, Truth Social. Some of these more recent social media platforms (especially Parler and Truth Social) were created specifically to combat what right-wing opinion leaders claimed to be a crackdown on free speech. On such platforms, hateful and prejudicial language may be entirely unfiltered, making them well-suited for empirical research into the connection between ideology and online prejudice.

## Concluding remarks

We believe that it is an appropriate time for social scientists to take stock and reflect on the question of how and why it is we study prejudice and discrimination in the first place. Initially, research in this area arose from the (belated) historical acknowledgement of exploitation and oppression faced by certain groups, such as racial, ethnic, religious, and sexual minorities, and perpetuated, generally speaking, by members of majority groups that were relatively high in social status, power, and material resources (e.g.,^9,31,49^). Many recent contributions to the debate about ideological symmetry vs. asymmetry in bias and prejudice are strikingly ahistorical and, it seems to us, lacking an appreciation of structural inequalities in society (e.g.,^21–28,42^). We contend that it is impossible to properly understand these phenomena without appreciating the significance of both longstanding and current imbalances of power and material resources in the overarching social system (e.g., see^5,48^). Our research program is offered as a wake-up call to those who would seek to strip the study of prejudice of its historical and social-structural origins in a naïve and, indeed, we would argue, ultimately futile attempt to de-politicize and “neutralize” the subject matter (see also^50^ for a similar critique of symmetrical approaches to the study of political polarization).

### Supplementary Information

Supplementary Information.

## Data Availability

Data from the human coding phase, as well as the final dataset from the machine learning phase are available via https://osf.io/6kj5s/?view_only=67175bcd980c444bbf47fb5b44dd8424.
