# Supplementary material for: Ideological asymmetries in online hostility, intimidation, obscenity, and prejudice
Source: Sci Rep. 2023 Dec 15;13:22345. doi: 10.1038/s41598-023-46574-2 (PMC10724124; doi:10.1038/s41598-023-46574-2)
Supplement: Supplementary file 1 — Supplementary Information. [file 41598_2023_46574_MOESM1_ESM.docx]

**Ideological Asymmetries in Online Hostility, Intimidation, Obscenity, and Prejudice**

**Supplementary Material**

**Supplement A**

**Human Coding Handbook**

**Project Objectives**

As part of this project, we aim to develop a tool/method to classify prejudicial language and hate speech online in a multifaceted manner.

In the first phase of the project, we will rely on human coding to rate thousands of Tweets on whether they contain several categories that we theoretically and empirically identified—by resorting to some key social psychological concepts as well as scanning through thousands of Tweets. Human coding is important for several reasons:

- It allows us to test for inter-rater reliability and gain confidence in the ratings obtained;
- It creates the basis for machine learning, where we use the input/ratings from human coders to train naïve AI classifiers to rate any other Tweets based on the code created. Human Coding

As research assistants on this project, you will handle the human coding element of the project. As such, each of you will be provided with:

- This coding manual as your go-to reference;
- An excel sheet containing [_____] randomly selected Tweets:
  - This sheet will contain columns for each of the categories you will be rating (see following section);
  - You will be rating the tweets in vivo, on the excel sheet itself.

We will run a pilot test on 500 randomly selected Tweets prior to coding the larger bulk of coding. This pilot will allow us to explore any limitations to our coding schema, suggestions to improve the coding process, and logistic issues. We will modify our procedure/codebook based on feedback from the pilot run.

**Coding Categories and Instructions**

- **Trigger Warning:**

Many of the Tweets that you will be coding will contain offensive language and possibly hate speech. Some of this language may pertain directly to social groups you strongly identify with. Be conscious that reading through some of the Tweets may not be a pleasant experience. These are publicly available data; therefore we have a chance to encounter them on a daily basis. If you are feeling stressed while reading the tweets, we encourage you to take a break to regroup. Please keep project leaders involved and come to them when and if you feel like your well-being is compromised while reading through any of the Tweets.

- **Objective Ratings:**

To reiterate, these Tweets have been collected based on a list of keywords of target groups of prejudice (across the political spectrum) that you may identify with. As such, it is important to remember to be an **objective rater**. Try to look at the Tweets from as objective of a lens as possible and rate the presence/absence of the linguistic categories regardless of the messages themselves or the groups that these messages are targeting. We want to be able to obtain accurate ratings across groups and across political ideology.

To ensure that, we will have sets of three research assistants rating the same set of Tweets, and then calculate interrater reliability. Make sure your ratings are accurate and unbiased. We aim to develop a scientific tool to assess prejudicial language online, and we want this to be a valid and reliable tool.

Occasionally, you may be unfamiliar with a word or phrase, but suspect that the word or phrase meets one of the criteria (e.g., obscenity, name-calling). If you suspect that the word or phrase meets the criteria for one of the categories, we suggest that you look up the definition of the word on Urban Dictionary or another online dictionary such as dictionary.com.

If a word that meets the criteria for a category is misspelled, the tweet should still be labeled as “1” for that category. For example, “fcuking” would be labeled as “Obscenity; Vulgarity”. If the word is censored (e.g., F******, $#!*), this **does not** meet the criteria for the category. If a term that would meet the criteria for a category is included as one of the words in an acronym (e.g., “IDGAF” = I don’t give a fuck), this also **does not** meet the criteria for the category.

- **Instructions:**

For each of the Tweets you have, you will have to rate for the presence/absence of each of the following pre-identified speech categories:

1. Threat; Intimidation; Violence
2. Obscenity; Vulgarity
3. Hate Speech; Racial, Ethnic, and Religious Slurs
4. Name-calling; Humiliation
5. ALL CAPS
6. Group-based stereotypes and generalizations
7. Negative Prejudice
8. Pornographic content
9. Non-English Language Tweets

The best way to go about this is by asking yourselves the leading question:

**“Is there any ____________ in this Tweet?”**

where the blank could be filled by each one of the categories consecutively.

- You should rate each of the categories as zero “**0**” for absence (answer to the leading question is ***no***), and one “**1**” for presence (answer to the leading question is ***yes***) of each of the categories.
- Remember that **these categories are not mutually exclusive**, and do not necessarily converge within each Tweet. As such, you could obtain a Tweet with threat, intimidation, or violence that is targeted toward a single individual on a personal basis, regardless of their group belonging. Such a Tweet would not contain prejudice. As you will see in the upcoming examples, Tweets in certain categories can easily belong to the other categories—that is totally acceptable. We are trying to look at the nuances of prejudicial language online instead of only classifying it as prejudice. As such, we find the definitions below necessary for understanding how we operationally define some of the “social psychological” coding categories.
- **Operational Definitions:**

**1. Threat; Intimidation; Violence:**

The World Health Organization (WHO) defines violence as “the intentional use of physical force or power, threatened or actual, against oneself, another person, or against a group or community, which either results in or has a high likelihood of resulting in injury, death, psychological harm, maldevelopment, or deprivation”. A tweet meets the criteria for this label if there is language threat containing a threat to use physical violence, or intimidation, which is [defined legally](https://definitions.uslegal.com/i/intimidation/) as “to make fearful or to put into fear” (i.e., scare tactics). Such threats of the use of violence or intimidation should be directed at an individual or a group.

**2. Obscenity, Vulgarity:**

An offensive word or phrase that is considered inappropriate to use in professional settings. A good heuristic to use is that if the word would be censored out of the “censored” version of the song, it is considered obscene or vulgar. Anything that would be, in PG terms, covered up by symbols to censor the language falls under this category as well (e.g., f**k or b@#%! or a**hole). Note that if the words **are censored through symbols, they do NOT get a rating of** 1. Only spelled out obscene or vulgar terms should get a rating of 1 for this category.

**3. Hate Speech; Racial, Ethnic, and Religious Slurs:**

Hate speech is a communication that carries no meaning other than the expression of hatred for some group. Racial and ethnic slurs are labels used to describe a racial, ethnic, or religious group that carry an inherently hateful and derogatory meaning. [Legally](https://definitions.uslegal.com/h/hate-speech/), hate speech is defined as:

“a communication that carries no meaning other than the expression of hatred for some group, especially in circumstances in which the communication is likely to provoke violence. It is an incitement to hatred primarily against a group of persons defined in terms of race, ethnicity, national origin, gender, religion, sexual orientation, and the like. Hate speech can be any form of expression regarded as offensive to racial, ethnic and religious groups and other discrete minorities or to women.”

**4. Name-calling; Humiliation:**

Language directed at an individual or group that is mocking, insulting, demeaning, or embarrassing. This includes dehumanizing language. Wikipedia’s entry on name-calling includes the following: “Name calling is abusive or insulting language referring to a person or group, a verbal abuse.” Examples could range from sissy or fag (as used in a derogatory manner, potentially related to group membership) to pig or ape or cow (comparisons to animals meant in a derogatory way).

**5. ALL CAPS:**

If **at least half of the tweet** is in ALL CAPS, the tweet should be labeled as ALL CAPS. If less than half of the tweet (e.g., one word) is in ALL CAPS, the tweet **should not** be labeled as ALL CAPS.

**6. Group-Based Stereotypes:**

According to Lawrence Blum (2004), “*stereotypes are false or misleading generalizations about groups held in a manner that renders them largely, though not entirely, immune to counterevidence*” (p. 251).

Examples of salient [cultural] stereotypes include (Blum, 2004, p. 252):

- Jews as greedy, wealthy, scholarly;
- Blacks as violent, musical, lazy, athletic, unintelligent;
- Women as emotional, nurturant, irrational;
- Asian-Americans and Asians as good at math and science, hardworking, a ‘model minority’;
- Irish as drinking too much;
- English as snooty;
- Poles as stupid; etc.

**7. Negative Prejudice:**

The essence of this work is to look at the manifestations of expressed prejudice in online speech, therefore we resort to the most classical, grounding definition of prejudice within social psychology.

The first, and still most prominent definition of prejudice came from Gordon Allport, who defined it as “*[a] feeling, favorable or unfavorable, toward a person or thing, prior to, or not based on, actual experience*” (Allport, 1954/1990, p. 6); more specifically, he operationalizes (negative) prejudice as “*an aversive or hostile attitude toward a person who belongs to a group, simply because he belongs to that group, and is therefore presumed to have the objectionable qualities ascribed to the group*” (p. 7).

Prejudice, thus, represents negative attitudes, or, in Allport’s terms, *an antipathy based on group-based generalizations and stereotypes* (Allport, 1954/1990; Dovidio, Hewstone, Glick, & Esses, 2010) [See above operational definition of stereotypes].

**8. Pornography:**

The purpose of this category is to label purely pornographic material and filter it out. Pornography is explicitly sexual material intended for sexual stimulation. If the tweet is pornographic, you should put a “1” for the pornography category and “0” for the other categories. This code should only be used if the material is clearly just advertisements for pornography. A tweet that contains sexual words, sexually objectifying language, or derogatory language with sexual overtones (e.g., using a word such as “cunt”, “fuck”, “rape”, or “whore”) is not necessarily pornographic, but may meet the criteria for other categories (e.g., obscenity, violence).

**9. Non-English Language Only:**

Any tweet that is entirely written in a non-English language. If the tweet is non-English only, you should put a “1” for the non-English only category and “0” for the other categories. This label should **NOT** be used if a tweet contains a mix of English and non-English language.

**References**

Allport, G. W. (1954/1990). The nature of prejudice. Reading, MA: Addison-Wesley.

Blum, L. (2004). Stereotypes and stereotyping: A moral analysis. Philosophical Papers, 33(3),

251-289.

**Supplement B**

**Intraclass Correlation Coefficients**

For all of the interrater reliability calculations, we used a two-way random-effects model, absolute agreement, ICC (2, *k*).

**Table S.1.** Interclass Correlation Coefficients for Dataset 1 (Pilot), *N* = 500 Tweets, 4 Raters

| **Category** | **ICC(2, *k*)** | **95% Confidence Interval** |
| --- | --- | --- |
| Threat | .822 | [.795, .846] |
| Obscenity | .810 | [.777, .839] |
| Name-calling | .701 | [.645, .748] |
| Hate speech | .702 | [.656, .743] |
| CAPS | .825 | [.799, .849] |
| Stereotypes | .719 | [.673, .760] |
| Negative prejudice | .765 | [.702, .813] |
| Porn | .694 | [.647, .735] |
| Non-English | .985 | [.983, .987] |

**Table S.2.** Interclass Correlation Coefficients for Dataset 2 (Pilot), *N* = 500 Tweets, 3 Raters

| **Category** | **ICC(2, *k*)** | **95% Confidence Interval** |
| --- | --- | --- |
| Threat | .466 | [.378, .543] |
| Obscenity | .500 | [.411, .577] |
| Name-calling | .553 | [.477, .620] |
| Hate speech | .751 | [.711, .787] |
| CAPS | .723 | [.678, .763] |
| Stereotypes | .746 | [.705, .782] |
| Negative prejudice | .775 | [.738, .807] |
| Porn | .857 | [.833, .877] |
| Non-English | .974 | [.970, .978] |

**Table S.3.** Interclass Correlation Coefficients for Dataset 3, *N* = 1000 Tweets, 4 Raters

| **Category** | **ICC(2, *k*)** | **95% Confidence Interval** |
| --- | --- | --- |
| Threat | .746 | [.713, .775] |
| Obscenity | .822 | [.802, .839] |
| Name-calling | .826 | [.807, .843] |
| Hate speech | .645 | [.601, .683] |
| CAPS | .797 | [.776, .817] |
| Stereotypes | .682 | [.637, .721] |
| Negative prejudice | .743 | [.696, .781] |
| Porn | .915 | [.906, .923] |
| Non-English | .990 | [.989, .991] |

**Table S.4.** Interclass Correlation Coefficients for Dataset, *N* = 1000 Tweets, 3 Raters

| **Category** | **ICC(2, *k*)** | **95% Confidence Interval** |
| --- | --- | --- |
| Threat | .646 | [.606, .682] |
| Obscenity | .806 | [.781, .828] |
| Name-calling | .604 | [.559, .644] |
| Hate speech | .535 | [.480, .580] |
| CAPS | .870 | [.856, .884] |
| Stereotypes | .435 | [.367, .489] |
| Negative prejudice | .497 | [.280, .539] |
| Porn | .831 | [.812, .849] |
| Non-English | .987 | [.986, .989] |

**Table S.5.** Interclass Correlation Coefficients for Dataset 5, *N* = 1000 Tweets, 3 Raters

| **Category** | **ICC(2, *k*)** | **95% Confidence Interval** |
| --- | --- | --- |
| Threat | .496 | [.438, .549] |
| Obscenity | .861 | [.845, .876] |
| Name-calling | .580 | [.532, .624] |
| Hate speech | .647 | [.607, .683] |
| CAPS | .866 | [.851, .880] |
| Stereotypes | .426 | [.297, .472] |
| Negative prejudice | .566 | [.445, .654] |
| Porn | .877 | [.863, .890] |
| Non-English | .980 | [.977, .982] |

**Table S.6.** Interclass Correlation Coefficients for Dataset 6, *N* = 1000 Tweets, 3 Raters

| **Category** | **ICC(2, *k*)** | **95% Confidence Interval** |
| --- | --- | --- |
| Threat | .429 | [.340, .477] |
| Obscenity | .886 | [.873, .898] |
| Name-calling | .513 | [.457, .564] |
| Hate speech | .709 | [.676, .739] |
| CAPS | .816 | [.795, .835] |
| Stereotypes | .336 | [.248, .414] |
| Negative prejudice | .469 | [.385, .541] |
| Porn | .748 | [.720, .774] |
| Non-English | .977 | [.975, .980] |

**Table S.7.** Interclass Correlation Coefficients for Dataset 7, *N* = 1000 Tweets, 3 Raters

| **Category** | **ICC(2, *k*)** | **95% Confidence Interval** |
| --- | --- | --- |
| Threat | .691 | [.656, .723] |
| Obscenity | .658 | [.617, .694] |
| Name-calling | .396 | [.318, .464] |
| Hate speech | .546 | [.495, .593] |
| CAPS | .785 | [.761, .807] |
| Stereotypes | .562 | [.506, .612] |
| Negative prejudice | .401 | [.320, .472] |
| Porn | .922 | [.914, .930] |
| Non-English | .978 | [.975, .980] |

**Table S.8.** Interclass Correlation Coefficients for Dataset 8, *N* = 1000 Tweets, 3 Raters

| **Category** | **ICC(2, *k*)** | **95% Confidence Interval** |
| --- | --- | --- |
| Threat | .213 | [.126, .293] |
| Obscenity | .792 | [.767, .814] |
| Name-calling | .672 | [.618, .717] |
| Hate speech | .704 | [.671, .735] |
| CAPS | .822 | [.802, .840] |
| Stereotypes | .391 | [.308, .464] |
| Negative prejudice | .532 | [.462, .591] |
| Porn | .983 | [.982, .985] |
| Non-English | .983 | [.981, .985] |

**Supplement C**

**Machine Learning Methodology and Results**

**Methods**

***Data***

The tweets were collected from the Twitter API using searches for certain keywords. These keywords correspond to groups that may be targets of hate speech and they include groups who are perceived as left-leaning and right-leaning, based on prior research. A total of 11 trained research assistants annotated a subset of the overall tweets.

There were 7k labeled tweets randomly selected from the 734k tweets and assigned to 3 or 4 annotators. The annotators were each trained by the same researchers using the same manual, but due to turnover, the same raters were not available for the entirety of the labelling process. We reserved 20% (1.4k) of the tweets as a test set to evaluate final performance. Of the remaining 5.6k tweets, 20% (1.1k) were used as validation, leaving ˜4.5k tweets for training. To create the labels, we relied on majority voting, where if 2 annotators agreed that the tweets contained hate speech, obscenity, etc. then it was labeled as belonging to the positive class. The prevalence of each class in the training data is summarized in the table below.

**Table S.9.** Prevalence of Each Linguistic Class in the Training Data

| **Label** | **Percentage** |
| --- | --- |
| Hate speech | 2.4% |
| Name calling | 7.3% |
| Negative prejudice | 13.3% |
| Non-English | 8.3% |
| Obscenity | 4.8% |
| Porn | 0.9% |
| Stereotypes | 8.0% |
| Threat | 2.8% |

***Text Processing Techniques***

In rare cases, the set contained identical tweets because of retweets, but because the retweets are not guaranteed to be annotated in an identical way, we preserved the each tweet in order to train the model on all available annotator choices. We experimented with two strategies for hyperlinks and @ mentions - removing them completely or replacing them with generic tokens ‘x user mention’ and ‘x url’. Other preprocessing choices were standard - lowercasing, tokenizing, and removing html remnants.

***Support Vector Machines***

Support Vector Machines is a popular supervised method for classification. In addition to adopting the text processing techniques above, we removed common stop-words from the corpus and then applied term frequency–inverse document frequency, a statistical method that adjusts the count occurrences of words based on the frequency within the different text documents. We explored different regularization techniques and created n-grams from size 1 to size 5.

***LSTM***

Our LSTM model is similar to the architecture described in Badjatiya et al. [1]. We initialized our embeddings from two sets of pre-trained vectors. The first is a a set of GLoVe vectors made available by Pennington et al [2]. We chose the embeddings pre-trained on 2B tweets for high domain relevance. The second is a set of vectors we trained on our own data, using open-source code [3]. We trained for 50 epochs on all 734k tweets in our data, with no minimum requirement for the number of times the word appears in the corpus. When the model initializes the embeddings, it first looks for each word among the publicly available pre-trained vectors, then, if not found, it finds the word among our own pre-trained vectors.

The model has one LSTM layer and a dropout layer with p = 0.20 for regularization.

***ULMFiT***

The ULMFiT model [4] was developed by researchers at fast.ai, a research institution dedicated to making deep learning more accessible. The ULMFiT model sought to apply techniques used in transfer learning for computer vision to the NLP discipline.

The ULMFiT model consists of three stages:

1. General-domain LM pre-training, which uses unlabeled data from a general domain.
2. Target task LM fine-tuning, which uses data from the same distribution as the labeled data, but which may be unlabeled.
3. Target task classifier fine-tuning, which uses the labeled data.

The general-domain language model is pretrained on Wikitext-103, which consists of 28,595 preprocessed Wikipedia articles.

Each phase of the model has a similar architecture, an LSTM-AWD [5]. The LSTM-AWD (ASGD Weight Dropped) is an LSTM with additional innovations that primarily serve to regularize the model.

The LSTM-AWD uses averaged stochastic gradient descent (ASGD) as its optimizer. Like SGD, ASGD computes a stochastic gradient at each step, but rather than returning the weights resulting from the last step, the algorithm returns an average of the weights over the last few steps. The interval over which to take an average is computed in a dynamic way depending on the validation perplexity.

The LSTM-AWD also uses weight-dropping, which differs from the most common way that dropout is applied. Dropout temporarily removes some randomly selected units from a network to promote independence rather than coadaptation between feature maps.

To differentiate between weight-dropping and a more traditional application of dropout, we review the LSTM architecture. The mathematical formulation of the LSTM is below:


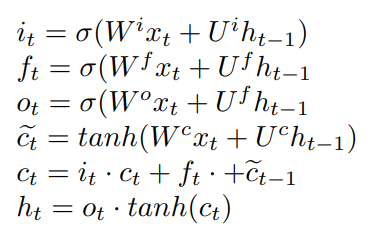


It is common for LSTM-based models to have a dropout layer that operates on the last hidden state of the LSTM *h_t_* prior to mapping the hidden layer to the outputs. Our standalone LSTM implements this strategy. Weight-dropping however, is dropout applied within the recurrent connections of the RNN. Weight-dropping acts on the hidden-to-hidden weight matrices within the LSTM, [*U^i^*, *U^f^*, *U^0^*, *U^c^*] in the mathematical formulation above. The ULMFiT model uses a 3-layer LSTM-AWD for each phase - language modeling, language fine-tuning, and text classification. The diagram below illustrates this.

**Figure S.1.** ULMFiT Diagram


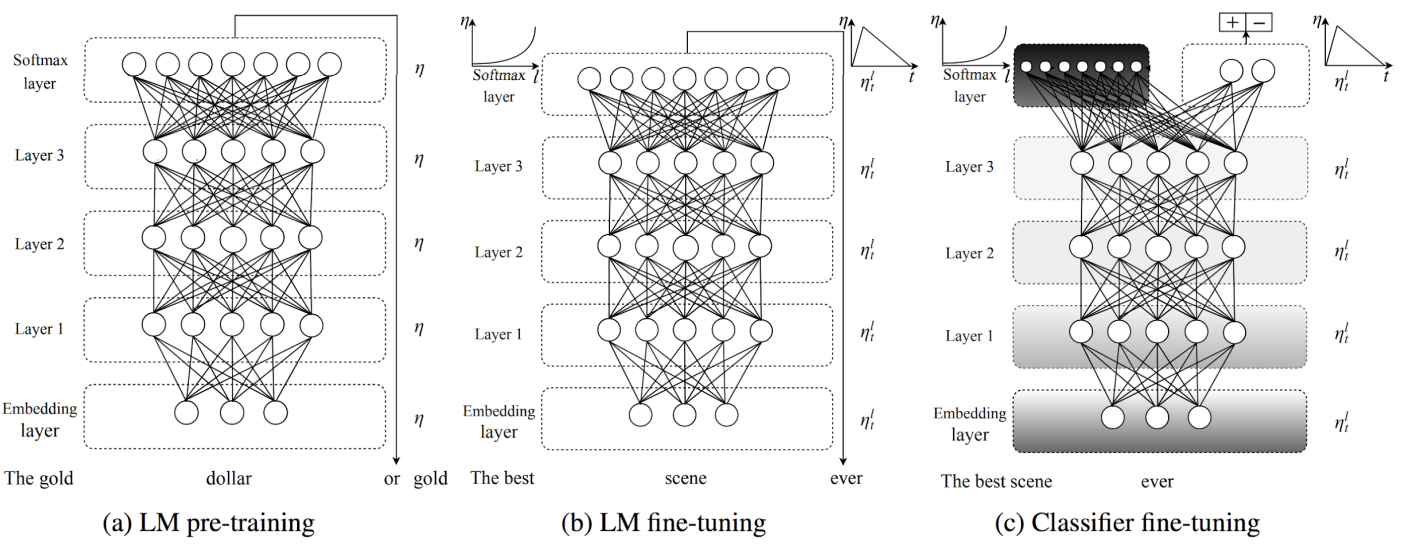


The ULMFiT model has been successfully applied to the problem of hate speech detection. Rother and Rettberg [6] applied ULMFiT to the problem of hate speech detection in a very similar configuration to our own. Using a dataset of only 5k labeled tweets, 303k unlabeled tweets, and the publicly available model pre-trained on Wikipedia data, they achieved an average validation f-score of 0.8. They also implemented a few improvements on Howard et. al’s original version of the ULMFiT model. They used a learning rate finder rather than relying on Howard et. al’s fixed “discriminative learning rate” schedule. They also greatly reduced the dropout during the language-medium model phase (testing multipliers of 0.7 and 0.3) and greatly increased it during the classifier stage, using a multiplier of 1.8. (Increasing the classifier dropout multiplier did not improve performance in our case.)

For our implmentation of the ULMFiT model, we used publicly available code released by the creators of ULMFiT [7]. We used the pre-trained language models available here. We ultimately used only the forward models. We fine-tuned the language model on all 734k tweets, before training the classifier on the labeled tweets.

***BERT***

Bidirectional Encoder Representations from Transformers or BERT is an innovative state-of-the-art language representation model [8]. Developed by researchers at Google AI Language, BERT creates a “deep bidirectional representation” of language - meaning that the representation of the language is contextualized, with each word conditioned on the words to its left and its right. A traditional language model is built by optimizing an objective function that seeks to accurately predict the next word, given the preceding context. BERT instead randomly “masks” words and seeks to predict the masked word given the context on the left and on the right.

The BERT model is “deeply bidirectional,” which differs from other similar language models. There is a bidirectional version of the ULMFiT model; it’s possible to pre-train both a forward and a backward language model, train two classifiers independently, and average the classifier prediction. (In our implementation, we didn’t use bi-directionality as it doubles the computational costs.) BERT, in contrast, creates one united bidirectional model rather than ensembling two independent models trained in each direction.

BERT’s authors contrast their approach to that of OpenAI GPT and ELMo. Though there are differences between the models, ELMo and ULMFiT both involve independently training left-to-right and right-to-left LSTMs. ELMo uses the concatenation of the LSTMs from each direction to generate features for downstream tasks. OpenAI GPT does not use bi-directionality.

**Figure S.2.** BERT Diagram


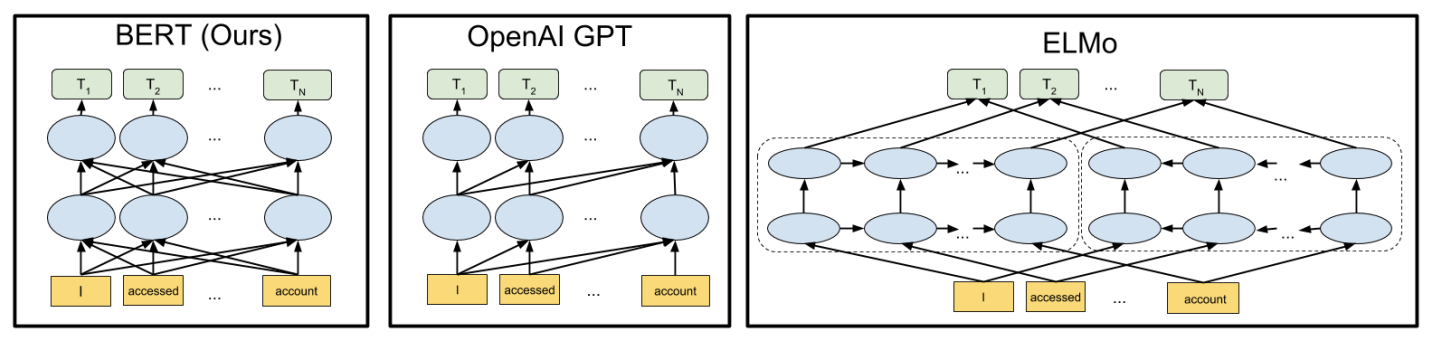


BERT is also pre-trained in such a way to build sentence and paragraph level comprehension. BERT is trained on the task of seeking to predict the next sentence. This particular innovation is probably not as crucial for classifying Twitter data due to its brevity, but BERT is intended to serve as a basis for a wide variety of NLP tasks.

BERT uses units call transformers, as originally implemented by Vaswani et al. [9]. The transformer is an alternative to convolutional and recurrent architectures that builds on the concept of multi-head attention. Traditional attention mechanisms in sequence-to-sequence models establish a correspondence between units of the input and units of the output. Multi-head attention can relate parts of a single sequence to each other, within either the input or the output.

The BERT model also represents language as word parts, not just full word tokens. So for example, it divides the word “mongering” into “mon” “ger” “ing.” This is not particularly novel, as techniques such as bipartite encodings of words have become common in NLP research, but it is especially relevant to Twitter data. Twitter data often contains misspellings and abbreviations, and hashtags are often comprised of several words combined without a space, so tokenizing only on words properly divided by spaces is limited.

Each sequence is represented as a series of token embeddings (the word or word part itself), segmentation embeddings (which identify which sentence the token belongs to), and position embeddings (which identify the order of the token within the sequence). Each sequence also begins with a special classification embedding, which is used for aggregate sequence representation.

We use BERT for sequence classification, which applies a classifier on top of the BERT Model. The classifier itself is extremely simple and shallow, relying extensively on the language understanding of BERT. The final hidden state of the first token in the special classification embedding provides a pooled representation of the sequence. The classifier applies dropout to this pooled representation, followed by a linear layer mapping between the result to the possible classes.

To implement our version of the BERT model, we used the publicly available PyTorch code. Although the original authors of BERT used TensorFlow, they have formally endorsed the PyTorch implementation, and experiments have verified that it produces identical results [10]. We start from the publicly available BERT model, pretrained on the BooksCorpus (800M words) and English Wikipedia (2,500M words). There are two publicly available versions of the BERT model, a large and a base. The large version has 16 attention heads and 24 layers, while the base version has 12 attention heads and 12 layers.

**Results**

***SVM***

Before exploring deep learning methods on the Twitter data, we hypertuned a few different SVM models as a first step to understand the relationship between the different speech labels. Table S.10 below represents our results. Overall, the SVM model produced sufficient results for the hate speech and obscenity categories, but performed poorly at predicting stereotypes, threats, and negative prejudice. The difference in performance between two different SVM models is partly due to sparse representation of our dataset, and L1 regularization typically performs better for such representations. This is evident in our results, as for most of the offensive speech categories, L1 outperformed L2 regularization.

**Table S.10.** SVM Tuning Results (*f*-Scores)

| **Label** | **SVM (L1 Penalty)** | **SVM (L2 Penalty)** |
| --- | --- | --- |
| Hate speech | .670 | .615 |
| Name calling | .144 | .335 |
| Negative prejudice | .262 | .367 |
| Non-English | .337 | .645 |
| Porn | .495 | .600 |
| Obscenity | .703 | .634 |
| Stereotypes | .169 | .221 |
| Threat | .144 | .198 |

***LSTM***

Below are the results of parameter tuning for the LSTM model, predicting hate speech. When we “remove” the mention and the url, we remove all @mentions and urls from the model. When we “replace” them, we use “x mention” and “x url” as tokens in place of the specific mentions and urls. Increasing the hidden size seems to be the most positively impactful hyperparameter choice.

**Table S.11.** Parameter Tuning for LSTM Model Predicting Hate Speech

| **Mention + url** | | **Seq Length** | **Vocab** | **Hidden Size** | **Batch Size** | **f1** |
| --- | --- | --- | --- | --- | --- | --- |
| Remove | 25 | | 10K | 200 | 32 | 0.615 |
| Remove | 30 | | 10K | 200 | 32 | 0.653 |
| Replace | 25 | | 10K | 200 | 32 | 0.640 |
| Replace | 30 | | 20K | 200 | 32 | 0.667 |
| Replace | 40 | | 10K | 200 | 32 | 0.682 |
| Replace | 40 | | 12K | 200 | 32 | 0.696 |
| Replace | 40 | | 12K | 400 | 16 | 0.744 |
| Replace | 40 | | 12K | 400 | 32 | 0.750 |

For the other labels, only the batch size was further tuned, using a model with a sequence length of 40, a vocabulary of 12k words, and a hidden size of 400. Results are presented in the below table.

**Table S.12.** Tuned LSTM Model Results (*f*-Scores)

| **Label** | **Batch Size 32** | **Batch Size 16** |
| --- | --- | --- |
| Hate speech | 0.750 | 0.744 |
| Name calling | 0.493 | 0.468 |
| Negative prejudice | 0.462 | 0.468 |
| Non-English | 0.920 | 0.941 |
| Porn | 0.615 | 0.727 |
| Obscenity | 0.500 | 0.667 |
| Stereotypes | 0.376 | 0.388 |
| Threat | 0.359 | 0.312 |

***ULMFiT***

Below are the results for tuning the ULMFiT model. The first two language models were trained with the default parameters. Then two more language models were trained with a larger vocabulary, a much shorter BPTT (backpropagation through time), and higher dropout. The back-propagation through time parameter controls how many words the model conditions on to predict the next word. The default value of 70 would usually span more than one tweet, so we shortened it to only 5 words. The classifier overfits quickly and more than 3 epochs tend to reduce performance. A lower learning rate is also helpful.

Table S.13 below summarizes the fine-tuned language models, and Table S.14 summarizes the trained classifiers.

**Table S.13.** Fine-tuned ULMFiT Models

| **ID** | **Dropout** | **Epochs** | **BPTT** | **Vocab** |
| --- | --- | --- | --- | --- |
| A | 1 | 20 | 70 | 30K |
| B | 1 | 35 | 70 | 30K |
| C | 0.70 | 20 | 10 | 60K |
| D | 0.70 | 20 | 5 | 60K |
| E | 0.70 | 7 | 5 | 60K |

**Table S.14.** Summary Results for Trained ULMFiT Classifiers (*f*-scores)

| **LM ID** | **Classifier ID** | **BPTT** | **Dropout** | **Epochs** | **Learning Rate** | **Batch** | **f1** |
| --- | --- | --- | --- | --- | --- | --- | --- |
| A | 1 | 70 | 1 | 1 | 0.01 | 64 | 0.640 |
| A | 2 | 70 | 1 | 2 | 0.01 | 64 | 0.642 |
| B | 3 | 70 | 1 | 3 | 0.01 | 64 | 0.667 |
| A | 4 | 70 | 1 | 5 | 0.01 | 64 | 0.654 |
| C | 5 | 40 | 1 | 3 | 0.01 | 64 | 0.667 |
| D | 6 | 40 | 1 | 3 | 0.01 | 64 | 0.702 |
| D | 7 | 40 | 1 | 4 | 0.01 | 64 | 0.610 |
| D | 8 | 40 | 0.70 | 3 | 0.01 | 64 | 0.655 |
| E | 9 | 40 | 0.70 | 3 | 0.005 | 64 | 0.681 |
| D | 10 | 40 | 0.70 | 3 | 0.005 | 64 | 0.634 |
| E | 11 | 40 | 0.70 | 3 | 0.005 | 32 | 0.710 |

Classifiers 5 and 6 are identical except for the bptt of the underlying language model. The classifier using language-model D, fine-tuned with a bptt of 5, performed better than the classifier using language model C, fine-tuned with a bptt of 10.

Classifiers 9 and 10 are identical except for the length of time that the underlying language model was trained. Language model D was trained for 20 epochs while language model E was trained for 7 epochs. Counter-intuitively, the language model trained for less time produced the higher-performing classifier.

We tuned primarily with respect to the hate speech label. The performance on all labels is presented in Table S.15 below. These results correspond to the parameters of classifier 11.

**Table S.15.** Performance of ULMFiT Classifier 11 Across Labels (*f-*scores)

| **Label** | ***f-*score** |
| --- | --- |
| Hate speech | 0.710 |
| Name calling | 0.307 |
| Negative prejudice | 0.326 |
| Non-English | 0.909 |
| Obscenity | 0.594 |
| Porn | 0.640 |
| Stereotypes | 0.258 |
| Threat | 0.364 |

***BERT***

Our most optimally performing classifier was BERT. Full results, including the tuning performed on the BERT models used for final classification, and the final test scores for the BERT model, are presented in the main text.

***Model Comparison***

The results of the best-performing model in each linguistic category are presented in Figure S.3 below.

**Figure S.3.** Model Comparison Across Categories


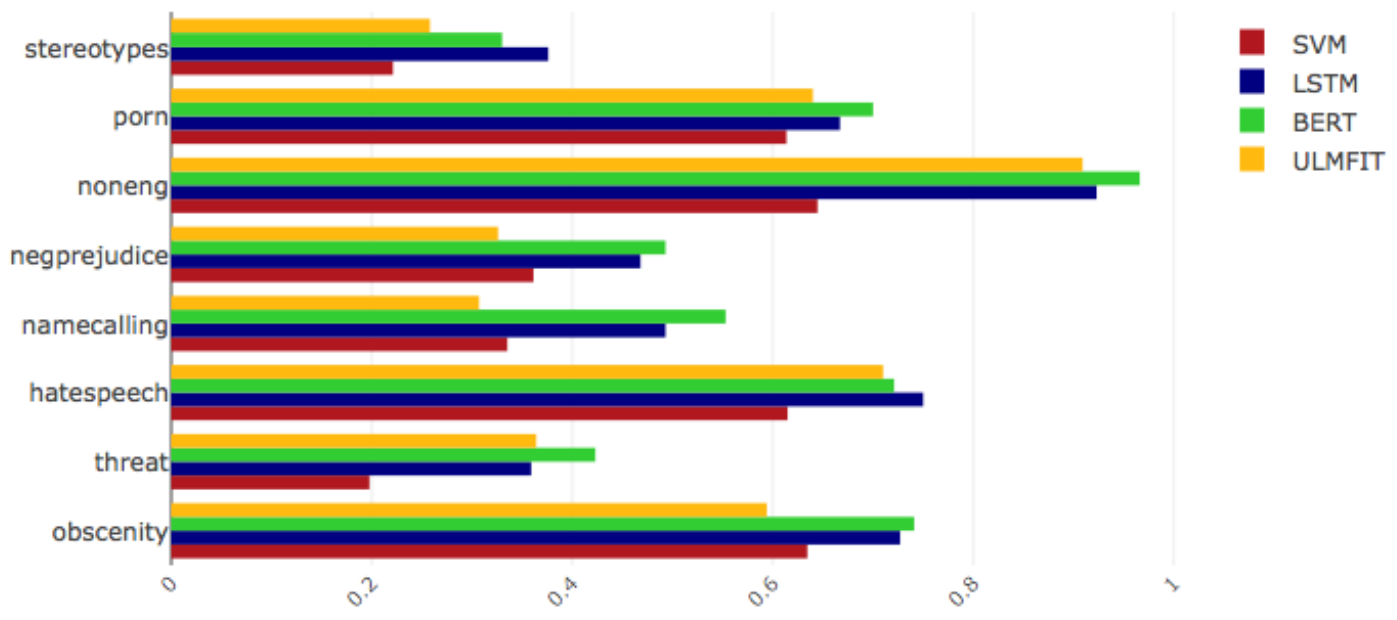


We make the following observations:

- We achieved significant improvement over our baseline model in every category.
- ULMFiT did not achieve the highest performance for any label.
- LSTM had the highest performance on hate speech.
- BERT had the highest performance on 6 of the 8 labels, and the highest average performance across labels.

BERT performs slightly lower than the LSTM on the hate speech and stereotypes labels, but it outperforms the LSTM on all other labels. The gains from transfer learning methods were somewhat modest. Twitter data differs greatly from the general domain datasets that ULMFiT and BERT were trained on.

Another consideration as it relates to the LSTM is that the f-score fluctuates greatly as it trains, and the f-scores reported for the LSTM are only the highest f-score observed during the training process. The performance of the LSTM is highly reliant on stopping training at the correct point. As currently implemented, early stopping prevents us from training on the validation set, and the dependence on accurate early stopping suggests that there may be a performance gap between the validation score and the test score.

In contrast, because the BERT model achieves strong performance with a fixed number of epochs, we expect to see less of a performance gap between the validation and test scores, and in fact we may see some improvement between the validation scores and the test scores from being able to also train on the validation data.

In summary, we chose BERT as our final model due to:

- Globally better performance than the SVM and ULMFiT on all labels.
- Better performance than the LSTM on almost all labels, including obscenity, name-calling, threat, and negative prejudice.
- Test scores from BERT may actually be higher than validation scores due to the opportunity to use more training data, without the need for a holdout set for early stopping.

**References**

[1] Pinkesh Badjatiya, Shashank Gupta, Manish Gupta, and Vasudeva Varma. Deep learning for hate speech detection in tweets. In *Proceedings of the 26th International Conference on World Wide Web Companion*, WWW ’17 Companion, pages 759–760, Republic and Canton of Geneva, Switzerland, 2017. International World Wide Web Conferences Steering Committee.

[2] Jeffrey Pennington, Richard Socher, and Christopher D Manning. Glove: Global vectors for word representation. In *EMNLP*, volume 14, pages 1532– 1543, 2014.

[3] Jeffrey Pennington, Richard Socher, and Christopher D Manning. Global vectors for word representation. https://github.com/stanfordnlp/ GloVe.

[4] Jeremy Howard and Sebastian Ruder. Universal language model fine-tuning for text classification. In *ACL*. Association for Computational Linguistics, 2018.

[5] Stephen Merity, Nitish Shirish Keskar, and Richard Socher. Regularizing and optimizing LSTM language models*. CoRR*, abs/1708.02182, 2017.

[6] Kristian Rother and Achim Rettberg. ULMFiT at germeval-2018: A deep neural language model for the classification of hate speech in German tweets. 09 2018.

[7] Sebastian Ruder. Imdb scripts. https://github.com/fastai/fastai/ tree/master/courses/dl2/imdb_scripts, 2018.

[8] Jacob Devlin, Ming-Wei Chang, Kenton Lee, and Kristina Toutanova. BERT: Pre-training of deep bidirectional transformers for language understanding. *arXiv preprint*:1810.04805, 2018.

[9] Ashish Vaswani, Noam Shazeer, Niki Parmar, Jakob Uszkoreit, Llion Jones, Aidan N. Gomez, Lukasz Kaiser, and Illia Polosukhin. Attention is all you need. *CoRR*, abs/1706.03762, 2017.

[10] Thom Wolf and Victor Sanh. PyTorch pretrained BERT. https://github. com/huggingface/pytorch-pretrained-BERT, 2018.
